# Prognostic and Clinicopathological Significance of Survivin in Colorectal Cancer: A Meta-Analysis

**DOI:** 10.1371/journal.pone.0065338

**Published:** 2013-06-03

**Authors:** Andreas Krieg, Thomas A. Werner, Pablo E. Verde, Nikolas H. Stoecklein, Wolfram T. Knoefel

**Affiliations:** 1 Department of Surgery (A), Heinrich-Heine-University and University Hospital Duesseldorf, Duesseldorf, Germany; 2 Coordination Centre for Clinical Trials, Heinrich-Heine-University and University Hospital Duesseldorf, Germany; IISER-TVM, India

## Abstract

Survivin/BIRC5 is a potentially interesting prognostic marker and therapeutic target in colorectal cancer (CRC). However, the available data on survivin expression in CRC are heterogeneous. Thus, to clarify the prognostic relevance of survivin in patients with CRC and its association with clinicopathological parameters we performed a meta-analysis. We screened PubMed and EMBASE for those studies that investigated the prognostic value of survivin and its association with clinicopathological parameters in CRC. Data from eligible studies were extracted and included into the meta-analyses using a random effects model. Electronical literature search identified 15 studies including 1934 patients with CRC mostly detecting survivin by immunohistochemistry (IHC). Pooled hazard ratios of 11 studies that performed survival analysis revealed a positive correlation between survivin expression and poor prognosis (HR 1.93; 95% CI: 1.55–2.42; *P*<0.00001; I^2^ = 23%). Subgroup analyses with respect to the detection method, HR estimation, global quality score and the country of origin in which the study was conducted supported the stability of this observation. In addition, meta-analyses revealed a significant association between expression of survivin and the presence of lymph node metastases (OR: 0.37; 95% CI: 0.19–0.75; I^2^ = 61%) or blood vessel invasion (OR: 0.50; 95% CI: 0.28–0.90; I^2^ = 0%). Expression of survivin indicates poor prognosis and a pro-metastatic phenotype and may be useful in identifying a subgroup of patients that could benefit from a targeted therapy against survivin in CRC.

## Introduction

As reported by the International Agency for Research on Cancer, colorectal cancer (CRC) causes about 608.000 deaths worldwide per year, making it the fourth leading cause of cancer-related deaths after lung, stomach and liver cancer [1]. The therapy for patients with UICC (Union for International Cancer Control) stage I and II is the primary resection according to oncological principles. Due to the good results, with a 5 -year cancer-specific survival rate of 90% for stage I and 80% for stage II tumors, respectively, there is no proof of benefit for the administration of any adjuvant chemotherapy in stage I and II patients [2], [3]. In contrast, patients with lymph node positive stage III clearly profit from adjuvant chemotherapy after surgical resection of the tumor. Current adjuvant treatment concepts in CRC include 5-Fluorouracil and Folinic acid in combination with Oxaliplatin according to the FOLFOX4 scheme [4]. However, a major issue remains to be an effective treatment of recurrent CRC and advanced tumor stages with distant metastases (UICC stage IV). During the last years, novel chemotherapeutic concepts focused on the development of targeted therapies, that improved overall survival in patients with CRC [5]. A basic principle in identifying suitable molecular targets is to profile tumors for potential molecular biomarkers that are associated with prognosis and tumor progression [6]. Accordingly, molecular targets such as vascular endothelial growth factor (VEGF), human epidermal growth factor receptors (EGFR) and others have been identified in CRC and are either already used in anticancer therapies or are under evaluation in clinical trials [5].

Altered expression of proteins with anti-apoptotic potential is known to modulate tumor cell viability and resistance to programmed cell death. In addition, the overexpression of anti-apoptotic proteins leads to resistance against conventional chemotherapy. In this context, recently the inhibitor of apoptosis protein (IAP) family was found not only to be overexpressed in malignant tumors, but also to be associated with a poor prognosis [7]. Arguably, one of the most extensively studied members of the IAP family is survivin that contains only one copy of a conserved domain called baculoviral IAP repeat (BIR) [8]. Functionally, survivin not only acts as antagonist of apoptotic cell death by inhibition of caspases in a complex with X-linked inhibitor of apoptosis protein (XIAP), but also as a regulator of mitosis [9], [10]. Interestingly, a survivin-XIAP complex promotes via TGF-beta activated kinase 1 binding protein 1 (TAB1)/TGF-beta activated kinase 1 (TAK1) and subsequent Nuclear Factor kappaB (NF-κB) activation tumor cell invasion and metastasis by activation of the cell motility kinases FAK (focal adhesion kinase) and Src (sarcoma) [11].

Under physiological conditions, survivin is expressed in proliferating foetal tissues, but not in the majority of differentiated adult tissues [8]. Analyses of human transcriptomes identified survivin to be one of the 40 genes that were expressed at elevated levels in cancer tissues but not in normal cells [12]. Consistent with these investigations, during the last decades many studies reported not only an increased expression of survivin in the most common human neoplasms such as non-small cell lung cancer, gastric cancer, colorectal cancer and liver cancer but also an association with poor prognosis [13], [14], [15], [16].

Previous studies have suggested that overexpression of survivin in CRC might serve as a prognostic factor but the direct relationship of survivin expression levels to clinicopathological variables and patients’ survival remains to be controversial. Therefore, we performed a systematic review of the literature and analyzed the role of survivin as prognostic and clinicopathological marker in CRC by meta-analysis.

## Materials and Methods

### Literature Search

A literature search via PubMed and EMBASE databases was conducted on November 21^st^, 2012 to find articles that assessed the role of survivin in CRC using the following keywords and text words: (1) colon or colonic or colorectal or rectal, and (2) cancer or carcinoma or tumor or neoplasm, and (3) survivin or BIRC5.

### Selection Criteria

All eligible articles that examined the relationship between the expression of survivin and clinicopathological variables and overall survival were extracted. Therefore, first the abstract and the title of the publications, which we received from our initial database analysis, were analyzed by A.K. to find exactly those articles that examined the association between survivin and clinicopathological parameters and/or overall survival in CRC. After the abstracts that met these criteria, were carefully read, the full texts were analyzed and included into the meta-analysis according to the following criteria: **(1)** expression of survivin was evaluated in CRC by immunohistochemistry or reverse transcription and polymerase chain reaction (RT-PCR) analysis; **(2)** expression levels of survivin were compared to patients clinicopathological characteristics and/or overall survival; **(3)** papers were written as full paper in English; **(4)** Hazard ratios (HR) for overall survival were provided or could be calculated from the data presented; **(5)** articles that provided sufficient data comparing the expression of survivin with clinicopathological data and that enabled us to calculate the Odds Ratio (OR); **(6)** if one author published data on the same group of patients in more than one journal, the most complete study was selected for our meta-analysis; **(7)** studies that provided only information about cytoplasmic and/or nuclear expression of survivin were excluded.

### Data Extraction

For data extraction, articles were reviewed by two independent investigators (A.K. and T.A.W.). Extracted data were recorded by both investigators independently in separate databases by including first author’s name, year of publication, study location, number of patients, gender, age, laboratory methodology, tumor characteristics, information about neoadjuvant therapy, cut-off value and HR with confidence interval (CI). Completed databases were compared and discussed by both investigators to find if required a consensus.

### Quality Assessment

Methodology quality was assessed by 2 independent investigators (A.K. and T.A.W.) by reading and scoring each publication according to the quality scale for biological prognostic factors established by the European Lung Cancer Working Party (ELCWP) [17]. This scale evaluates the scientific design, laboratory methodology, generalizability and results analysis. Each category can reach a maximum of 10 points, which theoretically results in a maximum total score of 40. Both investigators compared their calculated scores and, if necessary, achieved a consensus score for each category during a meeting. The final scores represent the percentage of the maximum achievable score, ranging from 0 to 100%. Thus, higher values reflect a better methodological quality.

Since category “results analysis” only allows the evaluation of articles that performed survival analyses, in this section it is impossible to evaluate the studies that have only examined the association of survivin with clinicopathological variables. In consequence, studies without survival analyses were characterized by a lower global score.

### Statistical Analysis

The strength of association between survivin positivity and clinicopathological parameters was expressed as OR. Clinicopathological variables included gender, depth of invasion, differentiation, lymph node metastases, lymphatic vessel invasion, blood vessel invasion and UICC stage or Duke’s classification. In some analyses data were combined, including T1 and T2 *versus* T3 and T4, UICC stage I and II (equivalent to Duke’s A and B) *versus* III and IV (equivalent to Duke’s C and D), or well and moderate differentiation *versus* poor differentiation. For this purpose, the number of survivin positive cases in relation to the total number of cases in each subgroup was subjected for the analysis of each variable.

HRs were used to describe the intensity of association between survivin expression levels and overall survival. An HR>1 indicated worse prognosis in patients with survivin overexpression. If HR and 95% CI were specified within the articles, these data were extracted and used to calculate the summarized HR. Otherwise, HR and 95% CI were estimated by reading Kaplan-Meier survival curves using the software Engauge Digitizer version 4.1 (http://digitizer.sourceforge.net/). Next, extracted data were utilized to reconstruct the HR and its variance by performing survival analysis (GraphPad Software, Inc, La Jolla, CA, USA), where we had to assume that the number of censored cases was constant during the period of follow-up.

Statistical heterogeneity was tested by Cochrane’s Q test (Chi-squared test; Chi^2^) and by measuring inconsistency (I^2^) [18], [19]. Since we had to assume that the data being analyzed consist of different populations, ORs and HRs with 95% CI were pooled by the DerSimonian and Laird method (random effects model) [20]. Stability of the meta-analysis was tested by subgroup and one-way sensitivity analyses. Review Manager 5.0 (http://ims.cochrane.org/revman) was used to perform meta-analysis and to prepare graphical results. Funnel blots were designed for assessing risk of publication bias. Non-parametric tests compared quality scores between distinct subgroups. A *P*-value less than 0.05 was considered to be statistically significant.

## Results

### Study Selection and Characteristics

**Figure 1:**
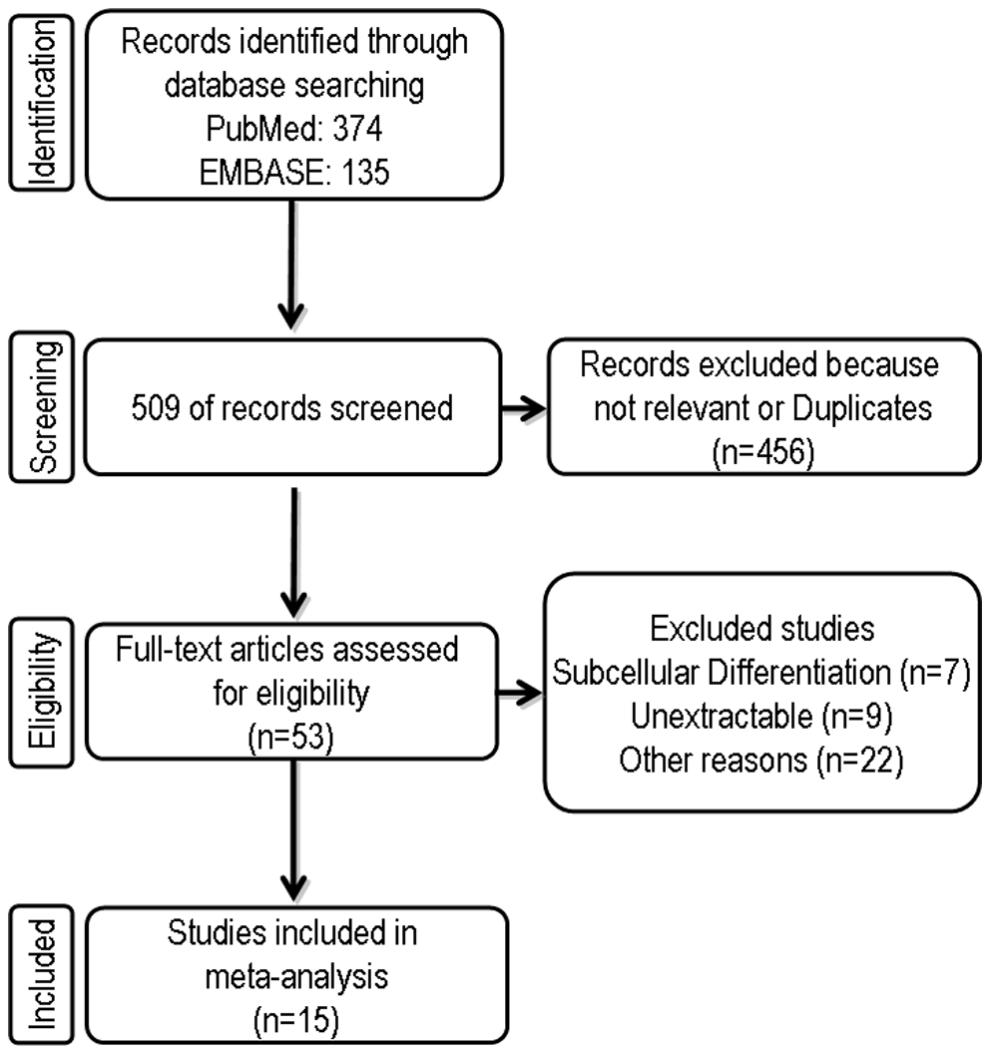
Flow chart summarising the literature search and study selection.

According to our defined criteria, electronic database search via PubMed and EMBASE retrieved 374 and 135 articles, respectively **(** **Figure 1** **)**. By careful reading the abstracts we identified 53 studies that focused on the expression of survivin in colon cancer specimen and thus were included in our full-text review process. After reading the full-text papers of the remaining 53 articles, 38 articles had to be excluded because they differentiated between survivin expression in the cytoplasm and nucleus (n = 7), data were not extractable (n = 9) or did not provide information about overall survival or clinicopathological parameters (n = 22). Finally, to evaluate the prognostic and clinicopathological significance of survivin as a potential biomarker in CRC we enrolled 15 eligible studies into our meta-analysis that were published between 1998 and 2012 [14], [21], [22], [23], [24], [25], [26], [27], [28], [29], [30], [31], [32], [33], [34].

As summarized in **table 1**, 10 studies included patients from Asia, 3 from Europe and 1 from Australia and Egypt, respectively. Expression of survivin was either detected by reverse transcriptase-polymerase chain reaction (RT-PCR) method (n = 2) or by immunohistochemistry (IHC; n = 13), whereas in 3 studies a tissue microarray (TMA) was constructed. Eleven studies enrolled patients with CRC of UICC stages I–IV, 1 study investigated survivin expression only in UICC stage III CRC and the remaining 3 studies did not provide any information regarding the UICC stage. Two studies included only patients with rectal cancer, and the majority of these patients received neoadjuvant radiotherapy (RT) or a combined radio-chemotherapy (RCT). One study reported only the inclusion of patients with colon cancer. Besides of the two above-mentioned publications, none of the other studies reported the use of neoadjuvant treatment concepts.

**Table 1:** Clinical and methodological characteristics of included studies.

First Author	Year	PMID	Country	Cases	Location	Stage	Neoadjuvant Therapy	Variables	Method	Cuttoff value	HR Estimate	HR	95% CI
Kawasaki	1998	9823313	Japan	171	CRC	I–IV	No	G,D,Du	IHC	5%	HR (MV)	0.86	0.42–1.77
Sarela	2000	10764707	UK	144	CRC	I–IV	NA	G,S	PCR	pos	HR (MV)	2.6	1.17–5.75
Lin	2003	12717841	China	87	CRC	NA	No	D	IHC	10%	NA	NA	NA
Knutsen	2004	15337550	Sweden	98	RC	I–IV	RT	G,D,Du	IHC	5%	HR (MV)	3.36	1.16–9.66
El-Hameed	2005	16353082	Egypt	230	CRC	I–IV	No	G,D,Du	TMA	5%	Sur. Curve (UV)	2.62	1.46–4.69
Hsiao	2006	16364925	Taiwan	41	CRC	III	NA	NA	IHC	>0%	Sur. Curve (UV)	3.13	1.14–8.62
Lam	2008	18547619	Australia	51	CRC	I–IV	NA	NA	PCR	5	Sur. Curve (UV)	3.84	1.53–9.67
Wang	2009	19728912	China	620	CC	I–IV	NA	NA	TMA	5%	HR (MV)	1.60	1.08–2.37
Lee	2009	19242064	China	95	CRC	I–IV	No	NA	TMA	≥180	HR (MV)	1.6	1.02–2.51
Liang	2009	19735100	China	100	CRC	I–IV	No	G,D,N,Du	IHC	10%	NA	NA	NA
Xiaoyuan	2009	19921309	China	68	CRC	NA	No	G,T,D,N	IHC	>0%	HR (MV)	1.99	1.17–3.38
Kalliakmanis	2010	20033843	Greece	77	CRC	I–IV	No	G,D,Du	IHC	5%	Sur. Curve (UV)	1.50	0.80–2.80
Xi	2011	21934342	China	61	CRC	I–IV	No	G,D,S	IHC	10%	Sur. Curve (UV)	2.70	1.07–6.84
Chu	2012	22065492	China	48	CRC	NA	No	N	IHC	>0%	NA	NA	NA
Takasu	2012	22936565	Japan	43	RC	I–IV	RCT	G,T,D,N,Lvi,Vi,S	IHC	5%	NA	NA	NA

Abbreviation: PMID, PubMed Id; G, gender; D, differentiation; S, UICC stage; Du, Dukès classification; T, depth of invasion; N; lymph node metastasis; Lvi; lymphatic vessel invasion; Vi, blood vessel invasion; NA, not available; IHC, immunohistochemistry, TMA, tissue microarray; PCR, polymerase chain reaction.

A total of 1934 patients were enrolled in the 15 studies (mean: 129; range: 41 to 620), whereas 11 studies including 1528 patients (mean: 139; range: 41 to 620) investigated the prognostic value of survivin in CRC. In 4 studies, data comparing clinicopathological parameters in the context of survivin expression were not reported or extractable. Six studies evaluated the association between survivin expression levels and overall survival by multivariate analysis, the remaining 5 presented survival curves.

### Study Quality

To estimate the quality of studies included into our meta-analysis, we evaluated study design, laboratory methodology, generalizability, results analysis and calculated a global quality score for each study. Then, the final global quality score was expressed as percentage to the maximum achievable total score. Hence, the mean global quality score of the included studies was 53.8% (range 37.5 to 72.5%) **(** **Table 2** **)**. However, it has to be considered that the score evaluates under the section “results analysis” only studies in which a survival analysis was performed. Consequently, since 4 studies did not provide survival data, they could not be scored in this category resulting in a low global quality score. Importantly, when comparing the quality scores for design, laboratory methodology and generalizability of publications presenting survival data with those analyzing only clinicopathological parameters, no statistically significant difference became obvious. Studies that performed a multivariate analysis achieved, as expected, a significant higher value for “results analysis” as well as for the global quality score. However, no significant difference in the quality of studies from Asia or other countries became evident.

**Table 2:** Study quality assessment according to the ELCWP Scale.

	No. of studies	Design	Laboratory methodology	Generalizability	Results analysis	Global Score (%)
All Studies	15	5.3	6.2	6	5.5	53.8
Survival Data	11	5.5	6.2	6	5.3	57.5
No Survival Data	4	5	6.3	6	NA	43.1
* P*-value		0.72	0.94	1.00	NA	0.02
HR	6	5.5	7	6.2	6.8	63.8
Sur. Curve	5	5.4	5.2	5.8	3.8	50.5
* P*-value		0.82	0.05	0.44	0.01	0.007
Asian	10	5.2	5.9	5.8	5.8	51
Other regions	5	5.6	6.8	6.4	5	59.5
* P*-value		0.25	0.28	0.31	0.52	0.17

Abbreviation: NA, not assessed.

### Study Results and Meta-analysis

First, we analyzed whether survivin expression levels were associated with the overall survival in patients with CRC. For this purpose, 11 studies with a total number of 1528 patients could be included. The majority of these studies analyzed the association between survivin and overall survival in all CRC stages. Sarela and colleagues [14] excluded stage IV tumors for survival analysis, and others investigated only stage III CRCs [25] or provided any information on tumor stage [30]. Out of these 11 studies, Kawasaki [21] and Kalliakmanis [31] found no significant association between survivin expression and overall survival. In addition, when using the HR and the *P*-value reported by Xiaoyuan [30], Review Manager 5.0 calculated differing CIs than those that were published.

**Figure 2:**
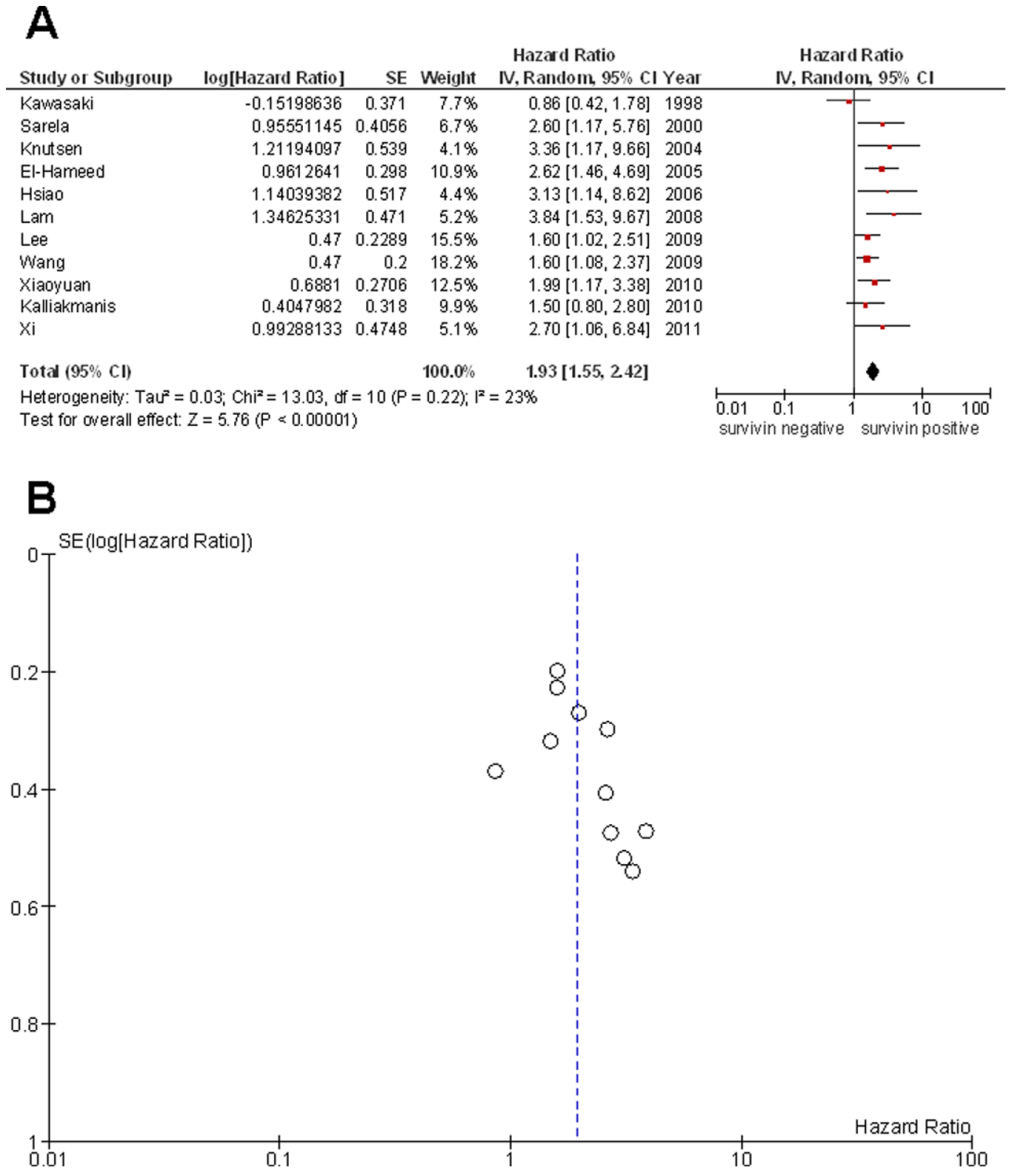
Meta-analysis comparing survivin expression and overall survival in CRC patients. (A) Forest blot reflects the individual and pooled HR with CI. Heterogeneity was calculated by the Cochrane Q test (Chi-squared test; Chi^2^) and by measuring the inconsistency (I^2^). (**B**) Funnel blot was designed to visualize a potential publication bias.

The pooled HR of all studies showed that high survivin expression levels were associated with a decrease in overall survival in CRC (HR 1.93; 95% CI: 1.55–2.42; *P*<0.00001) **(** **Figure 2** **A)**. Importantly, Cochrane Q test (Chi^2^ = 13.03; *P* = 0.22) and test of inconsistency (I^2^ = 23%) could exclude a significant heterogeneity. Moreover, when visually inspecting the funnel plot a publication bias became not obvious **(** **Figure 2** **B)**. In addition, we performed one-way sensitivity analysis by stepwise excluding a single study and calculating again the summarized HR for the remaining studies (data not shown). By this analysis, we underlined that the stability of our results supporting survivin as a prognostic marker in CRC were not influenced by any certain study.

Next, we performed subgroup analyses to investigate if there were differences in results with respect to the detection method, HR estimation, global quality score and the country of origin in which the study was conducted **(** **Table 3** **)**. Despite the limited number of studies that were eligible for this meta-analysis, the detection method for survivin had no impact on the prognostic value of survivin in CRC, although studies using RT-PCR analyses exhibited a more pronounced prognostic effect. Both, studies performing multivariate or univariate analyses found survivin to be a prognostic marker in CRC, although the latter was characterized by a higher pooled HR. After calculating the mean global quality score and categorizing studies into a group, which was characterized by a quality score that was higher or lower than the mean value, a conspicuous difference between both groups was not detected. Subgroup analyses regarding the country of origin of the study revealed that studies outside from Asia showed a stronger prognostic value for survivin in CRC. Importantly, all of these subgroup analyses revealed no heterogeneity. Although we could not perform a subgroup analysis on the definition of tumor stage, we calculated the summary HR for studies that enrolled patients with all disease stages I–IV (n = 8) and excluded studies that either did not specify the tumor stages or that included only certain tumor stages. However, the pooled HR for studies that included all tumor stages again supported survivin as a prognostic marker (HR: 1.85; 95% CI: 1.39–2.46; *P*<0.0001; I^2^ = 37%). The same prognostic value became obvious when we analyzed studies in which the text emerged that no concepts of neoadjuvant treatment were used (n = 8; HR: 1.80; 95% CI: 1.40–2.31; *P*<0.00001; I^2^ = 29%). When excluding studies that investigated only cancers located either in the rectum (n = 1) or colon (without rectum; n = 1), the pooled HR underlined once more the robustness of our results (HR: 1.98; 95% CI: 1.51–2.58; *P*<0.00001; I^2^ = 28%).

**Table 3:** Subgroup analyses evaluating methodological and demographic effects on the association between survivin and overall survival in CRC.

		Pooled Data (Random)				Test for Heterogeneity				
Subgroup	No. ofStudies	Cases	OR	95% CI	*P*-value	Chi^2^	*P*-value		I^2^ (%)	
*Method*										
IHC/TMA	9	1351	1.81	1.44–2.27	<0.00001	9.83	0.28		19	
PCR	2	177	3.07	1.68–5.61	0.0003	0.40	0.53		0	
*Survival analysis*										
Surv. Curve	5	350	2.40	1.71–3.36	<0.00001	3.60	0.46		0	
HR	6	1178	1.70	1.30–2.24	0.0001	6.58	0.25		24	
*Global Quality Score*										
≥57.5	5	558	1.76	1.21–2.57	0.003	6.47	0.17		38	
<57.5	6	970	2.09	1.56–2.79	<0.00001	5.94	0.31		16	
*Country*										
Asian	6	1056	1.69	1.29–2.20	0.0001	6.21	0.29		19	
Others	5	472	2.41	1.73–3.35	<0.00001	3.70	0.45		0	

**Figure 3:**
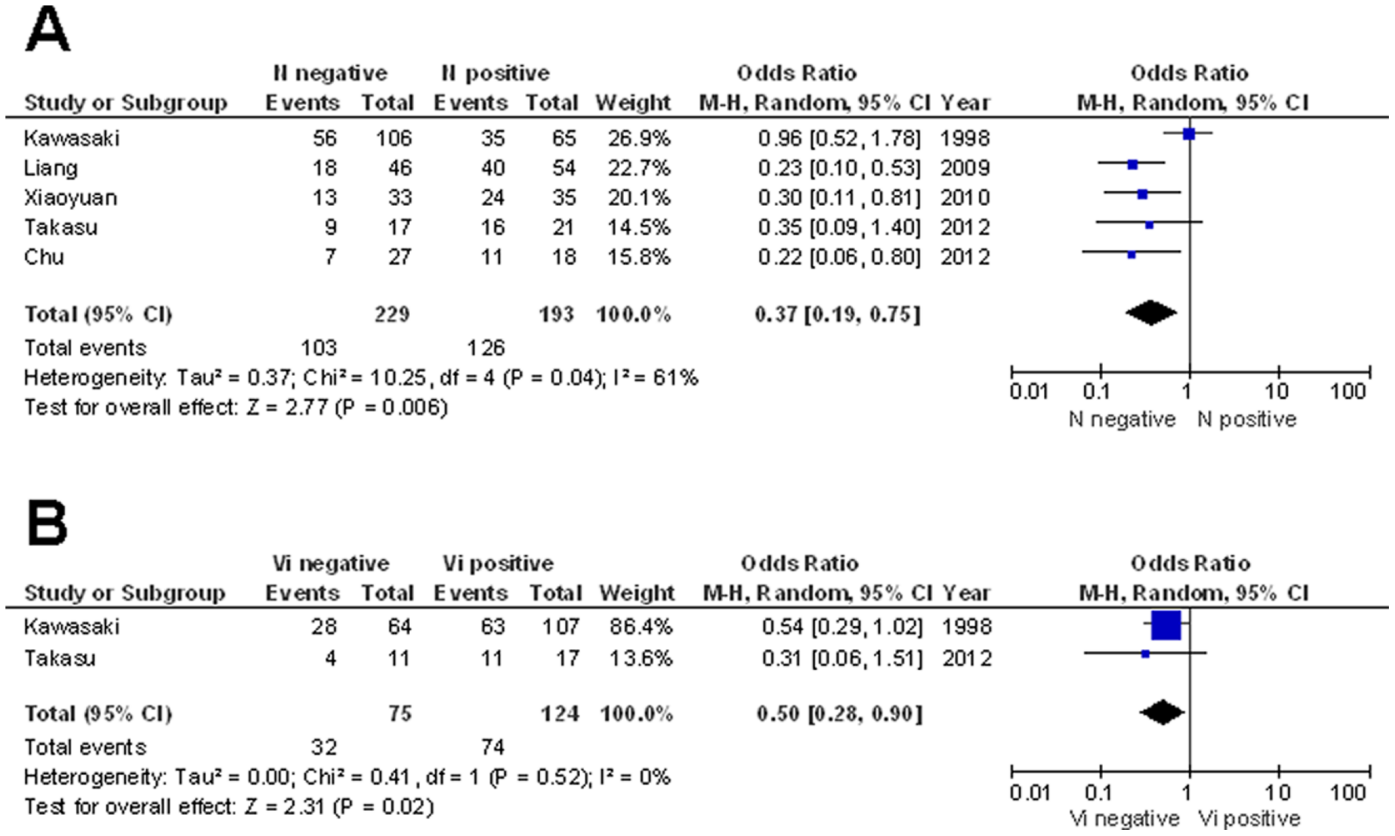
Association between survivin and lymph node metastasis or blood vessel invasion. Forest blot reflects the individual summarized OR with CI for the relationship between expression of survivin and (**A**) lymph node metastasis or (**B**) blood vessel invasion. Heterogeneity was verified by the Cochrane Q test (Chi-squared test; Chi^2^) and inconsistency (I^2^).

To gain further insights into the role of survivin as biological marker, we next investigated the association of survivin overexpression with clinicopathological parameters **(** **Table 4** **)**. Despite the limited number of studies, a random effect model revealed an association between expression of survivin and the presence of lymph node metastases or blood vessel invasion **(** **Figure 3** **A and B)**. Thus, the number of patients with positive lymph node status (OR: 0.37; 95% CI: 0.19–0.75; *P* = 0.006; I^2^ = 61%) or invasion of blood vessels by tumor cells (OR: 0.50; 95% CI: 0.28–0.90; *P* = 0.02; I^2^ = 0%) was higher in the group of survivin positive tumors. However, meta-analysis of studies investigating the relationship between survivin and lymph node metastasis showed a slight heterogeneity.

**Table 4:** Meta-analysis assessing the relationship between survivin expression and clinicopathological variables.

		Pooled Data (Random)				Test for Heterogeneity		
Clinicopathological Variable	No. of Studies	Cases	OR	95% CI	*P*-value	Chi^2^	*P*-value	I^2^ (%)
Gender (male/female)	9	992	1.15	0.88–1.49	0.32	5.80	0.67	0
UICC stage (I+II/III+IV)	8	924	0.70	0.48–1.01	0.06	11.24	0.13	38
Depth of invasion (T1+2/T3+4)	3	270	0.90	0.54–1.50	0.69	0.13	0.94	0
Differentiation (well+moderate/poor)	9	890	0.93	0.56–1.53	0.76	14.57	0.07	45
Lymph Node Metastasis	5	422	0.37	0.19–0.75	*0.006*	10.25	0.04	61
Lymphatic vessel invasion	2	209	1.95	0.91–4.17	0.08	0.70	0.40	0
Blood vessel invasion	2	199	0.50	0.28–0.90	*0.02*	0.41	0.52	0

## Discussion

Since Survivin/BIRC5, the smallest member of the IAP-family which is structurally characterized by only a single BIR domain, has been identified and demonstrated to be overexpressed in cancer tissues, it has attracted increasing interest [8]. Functionally, survivin exhibits distinct functions during cell cycle progression or as inhibitor of programmed cell death together with IAP-family member XIAP, by promoting stability of XIAP and synergistically inhibition of caspase-9 [10]. In addition, survivin promotes by complexing XIAP invasion and migration of malignant cells via NF-κB pathways, apparently contributing to metastasis [11]. As a consequence, over the last decades survivin has generated considerable interest as therapeutical target in cancer resulting in many studies that investigated the expression of survivin in malignant tumors, such as colon cancer, lung cancer, gastric cancer, renal cell carcinoma and many others [13], [15], [21], [35], [36]. However, some of these publications present conflicting data, even if they were performed in the same tumor entity. Thus, it is important to combine and investigate these data in meta-analyses to obtain a better understanding of a potential association between survivin and clinicopathological parameters as well as prognosis in cancer patients.

Accordingly, we conducted a meta-analysis of 15 eligible studies to evaluate the association between the expression of survivin and clinicopathological parameters or overall survival in patients with CRC. Interestingly, we found that survivin expression correlated with blood vessel invasion and the existence of lymph node metastases. The small number of studies that investigated the relationship between survivin expression and blood vessel invasion or lymph node metastasis might explain the heterogeneity that became obvious when comparing nodal status with survivin overexpression. On the other hand heterogeneity might be also explained by a variable extensiveness of lymphadenectomy during surgery among the studies or the use of different classification systems. Thus, none of the studies reported the number of lymph nodes that were retrieved by lymphadenectomy. In this context it has been suggested that the number of dissected lymph nodes predicts more precisely the survival in CRC patients which led to the recommendation to evaluate at least a minimum of 12 lymph nodes [37], [38].

In theory, the observation that overexpression of survivin was associated with blood vessel invasion and lymph node metastasis is supported by the results from Mehrotra and colleagues [11] that found survivin to stimulate tumor cell invasiveness as well as the formation of metastases in a complex with XIAP. Thus it is tempting to speculate that tumors expressing both survivin and XIAP might be characterized by a pronounced invasiveness and metastatic capacity.

In addition, our meta-analysis showed that expression of survivin was significantly associated with overall survival in CRC patients when analyzing survival data from 11 eligible studies including at total number of 1528 patients. Most importantly, when analyzing the prognostic significance of survivin in CRC we could exclude a serious heterogeneity.

However, our meta-analysis might have some limitations. One limitation is that we performed a search of databases that include only studies that have been published and that might not reflect representative populations because studies with positive results are more likely to be published than those representing negative data. In addition to this publication bias, we have to admit that a possible bias may be the fact that all studies were of retrospective nature, whereas, to the best of our knowledge, high quality randomized, controlled trials investigating the association of survivin with clinicopathological parameters or overall survival have not been published so far. Moreover, we included studies with different detection methods by using RT-PCR or immunohistochemistry, wherein, for the latter several different antibodies were used. However, subgroup analysis on the definition of IHC *versus* RT-PCR revealed only a stronger prognostic value in studies that performed RT-PCR analysis. Another source of bias might be due to the extraction of data from survival curves. The resulting HRs have to be considered to be less accurate than HRs from studies that provided results from multivariate analyses. Nevertheless, when we calculated the pooled HR only from multivariate datasets, the relationship between survivin and overall survival was still significant. We cannot exclude that we introduced a language bias by including only English written articles which might favour positive results [39].

Furthermore, we excluded 7 studies that differentiated between expression patterns of survivin within the nucleus or the cytoplasm, because only three of these studies provided survival data even with conflicting results. Although survivin has been demonstrated to be expressed in cancer cells nuclei and cytoplasm, the results are inconsistent which might be explained by the way of tissue-processing conditions or the existence of distinct splice variants in different subcellular compartments [40]. In this context, Mahotka et al. demonstrated a preferentially cytoplasmic location for survivin and survivin-2B, whereas a cell-cycle dependent nuclear distribution was found for survivin-deltaEx3 [41]. Moreover, the cytoplasmic pool of survivin has been suggested to be involved in the suppression of anoikis in CRC, a process promoting cancer cell survival during extravasation and invasion within the metastatic process [42]. Although we excluded studies that differentiated between the subcellular expression levels of survivin due to the small number, it has to be mentioned that Qi and colleagues found intracellular localization of survivin to determine biological behaviour in colorectal cancer [43]. Thus, in the future additional studies should be conducted addressing the importance of the subcellular localization of survivin as prognostic marker in CRC.

However, our data are consistent with meta-analyses supporting survivin as a prognostic marker in esophageal and non-small cell lung cancer [44], [45], [46]. In contrast to these meta-analyses, we additionally analyzed the association of survivin with clinicopathological parameters. Undoubtedly, in the future these results should be confirmed by prospective and randomized studies, but they provide new insights that support survivin as a potential prognostic biomarker and biological target for anticancer therapies in CRC.

The role of survivin in CRC is once more supported by the observation that survivin has been identified as a target of the APC/TCF/beta-catenin signalling pathway by this promoting a dysbalance between proliferation and apoptosis in the basal crypts during tumorigenesis [47], [48], [49].

During the last decades, many efforts have been made to develop survivin antagonists as targeted therapy in cancer. To date, first phase II trials using the survivin antagonist YM155, a small molecule that acts by inhibiting survivin promoter activity, was well tolerated by patients with prostate cancer and diffuse large B-cell lymphoma proposing this compound to be combined with other cytotoxic drugs [50], [51], [52]. In addition, Idenoue and colleagues reported a potent immunogenic cancer vaccine that targets survivin [53]. Thus, tumors with a particularly high expression of survivin might be a suitable target for anti-survivin-immunotherapies. In the future, it may be helpful to examine survivin expression in CRC specimens, thereby identifying patients that are characterized by a poor prognostic feature and that might benefit from targeted therapies against survivin even independently of their tumor stage.

In conclusion, our meta-analysis provides evidence that expression of survivin is associated with overall survival and a metastatic phenotype in patients with CRC. Thus, high survivin expression levels not only predict prognosis, but also may be useful in identifying a subgroup of patients that could benefit from a targeted therapy against survivin in CRC.

## References

[1] Ferlay J, Shin HR, Bray F, Forman D, Mathers C, et al. Estimates of worldwide burden of cancer in 2008: GLOBOCAN 2008. Int J Cancer 127: 2893–2917. 10.1002/ijc.25516 21351269

[2] Chok KS, Law WL (2007) Prognostic factors affecting survival and recurrence of patients with pT1 and pT2 colorectal cancer. World J Surg 31: 1485–1490. 17510767 10.1007/s00268-007-9089-0

[3] Gill S, Loprinzi CL, Sargent DJ, Thome SD, Alberts SR, et al (2004) Pooled analysis of fluorouracil-based adjuvant therapy for stage II and III colon cancer: who benefits and by how much? J Clin Oncol 22: 1797–1806. 15067028 10.1200/JCO.2004.09.059

[4] de Gramont A, Tournigand C, Andre T, Larsen AK, Louvet C (2006) Targeted agents for adjuvant therapy of colon cancer. Semin Oncol 33: S42–45. 17178286 10.1053/j.seminoncol.2006.10.006

[5] El Zouhairi M, Charabaty A, Pishvaian MJ (2011) Molecularly targeted therapy for metastatic colon cancer: proven treatments and promising new agents. Gastrointest Cancer Res 4: 15–21. 21464866 PMC3070284

[6] Bacolod MD, Barany F (2011) Molecular profiling of colon tumors: the search for clinically relevant biomarkers of progression, prognosis, therapeutics, and predisposition. Ann Surg Oncol 18: 3694–3700. 21347779 10.1245/s10434-011-1615-5 PMC4382964

[7] de Almagro MC, Vucic D (2012) The inhibitor of apoptosis (IAP) proteins are critical regulators of signaling pathways and targets for anti-cancer therapy. Exp Oncol 34: 200–211. 23070005

[8] Ambrosini G, Adida C, Altieri DC (1997) A novel anti-apoptosis gene, survivin, expressed in cancer and lymphoma. Nat Med 3: 917–921. 9256286 10.1038/nm0897-917

[9] Skoufias DA, Mollinari C, Lacroix FB, Margolis RL (2000) Human survivin is a kinetochore-associated passenger protein. J Cell Biol 151: 1575–1582. 11134084 10.1083/jcb.151.7.1575 PMC2150675

[10] Dohi T, Okada K, Xia F, Wilford CE, Samuel T, et al (2004) An IAP-IAP complex inhibits apoptosis. J Biol Chem 279: 34087–34090. 15218035 10.1074/jbc.C400236200

[11] Mehrotra S, Languino LR, Raskett CM, Mercurio AM, Dohi T, et al. IAP regulation of metastasis. Cancer Cell 17: 53–64. 20129247 10.1016/j.ccr.2009.11.021 PMC2818597

[12] Velculescu VE, Madden SL, Zhang L, Lash AE, Yu J, et al (1999) Analysis of human transcriptomes. Nat Genet 23: 387–388. 10.1038/70487 10581018

[13] Monzo M, Rosell R, Felip E, Astudillo J, Sanchez JJ, et al (1999) A novel anti-apoptosis gene: Re-expression of survivin messenger RNA as a prognosis marker in non-small-cell lung cancers. J Clin Oncol 17: 2100–2104. 10561264 10.1200/JCO.1999.17.7.2100

[14] Sarela AI, Macadam RC, Farmery SM, Markham AF, Guillou PJ (2000) Expression of the antiapoptosis gene, survivin, predicts death from recurrent colorectal carcinoma. Gut 46: 645–650. 10764707 10.1136/gut.46.5.645 PMC1727921

[15] Lu CD, Altieri DC, Tanigawa N (1998) Expression of a novel antiapoptosis gene, survivin, correlated with tumor cell apoptosis and p53 accumulation in gastric carcinomas. Cancer Res 58: 1808–1812. 9581817

[16] Ikeguchi M, Ueda T, Sakatani T, Hirooka Y, Kaibara N (2002) Expression of survivin messenger RNA correlates with poor prognosis in patients with hepatocellular carcinoma. Diagn Mol Pathol 11: 33–40. 11854600 10.1097/00019606-200203000-00007

[17] Steels E, Paesmans M, Berghmans T, Branle F, Lemaitre F, et al (2001) Role of p53 as a prognostic factor for survival in lung cancer: a systematic review of the literature with a meta-analysis. Eur Respir J 18: 705–719. 11716177 10.1183/09031936.01.00062201

[18] Lau J, Ioannidis JP, Schmid CH (1997) Quantitative synthesis in systematic reviews. Ann Intern Med 127: 820–826. 9382404 10.7326/0003-4819-127-9-199711010-00008

[19] Higgins JP, Thompson SG (2002) Quantifying heterogeneity in a meta-analysis. Stat Med 21: 1539–1558. 12111919 10.1002/sim.1186

[20] DerSimonian R, Laird N (1986) Meta-analysis in clinical trials. Control Clin Trials 7: 177–188. 3802833 10.1016/0197-2456(86)90046-2

[21] Kawasaki H, Altieri DC, Lu CD, Toyoda M, Tenjo T, et al (1998) Inhibition of apoptosis by survivin predicts shorter survival rates in colorectal cancer. Cancer Res 58: 5071–5074. 9823313

[22] Lin LJ, Zheng CQ, Jin Y, Ma Y, Jiang WG, et al (2003) Expression of survivin protein in human colorectal carcinogenesis. World J Gastroenterol 9: 974–977. 12717841 10.3748/wjg.v9.i5.974 PMC4611408

[23] Knutsen A, Adell G, Sun XF (2004) Survivin expression is an independent prognostic factor in rectal cancer patients with and without preoperative radiotherapy. Int J Radiat Oncol Biol Phys 60: 149–155. 15337550 10.1016/j.ijrobp.2004.02.007

[24] Abd El-Hameed A (2005) Survivin expression in colorectal adenocarcinoma using tissue microarray. J Egypt Natl Canc Inst 17: 42–50. 16353082

[25] Hsiao HL, Wang WS, Chen PM, Su Y (2006) Overexpression of thymosin beta-4 renders SW480 colon carcinoma cells more resistant to apoptosis triggered by FasL and two topoisomerase II inhibitors via downregulating Fas and upregulating Survivin expression, respectively. Carcinogenesis 27: 936–944. 16364925 10.1093/carcin/bgi316

[26] Lam AK, Saleh S, Smith RA, Ho YH (2008) Quantitative analysis of survivin in colorectal adenocarcinoma: increased expression and correlation with telomerase activity. Hum Pathol 39: 1229–1233. 18547619 10.1016/j.humpath.2008.01.001

[27] Wang GQ, Lu ZH, Fang YJ, Chen G, Zhou ZW, et al (2009) [Expression and clinical significance of survivin and matrix metalloproteinase-7 in colon cancer]. Ai Zheng 28: 945–949. 19728912 10.5732/cjc.008.10811

[28] Lee YY, Yu CP, Lin CK, Nieh S, Hsu KF, et al (2009) Expression of survivin and cortactin in colorectal adenocarcinoma: association with clinicopathological parameters. Dis Markers 26: 9–18. 19242064 10.3233/DMA-2009-0598 PMC3833605

[29] Liang QL, Wang BR, Li GH (2009) DcR3 and survivin are highly expressed in colorectal carcinoma and closely correlated to its clinicopathologic parameters. J Zhejiang Univ Sci B 10: 675–682. 19735100 10.1631/jzus.B0920077 PMC2738837

[30] Xiaoyuan C, Longbang C, Jinghua W, Xiaoxiang G, Huaicheng G, et al (2012) Survivin: a potential prognostic marker and chemoradiotherapeutic target for colorectal cancer. Ir J Med Sci 179: 327–335. 10.1007/s11845-009-0448-8 19921309

[31] Kalliakmanis JG, Kouvidou C, Latoufis C, Kouvatseas G, Anagnostakis D, et al (2011) Survivin expression in colorectal carcinomas: correlations with clinicopathological parameters and survival. Dig Dis Sci 55: 2958–2964. 10.1007/s10620-009-1088-6 20033843

[32] Xi RC, Biao WS, Gang ZZ (2011) Significant elevation of survivin and livin expression in human colorectal cancer: inverse correlation between expression and overall survival. Onkologie 34: 428–432. 21934342 10.1159/000331132

[33] Chu XY, Chen LB, Wang JH, Su QS, Yang JR, et al (2013) Overexpression of survivin is correlated with increased invasion and metastasis of colorectal cancer. J Surg Oncol 105: 520–528. 10.1002/jso.22134 22065492

[34] Takasu C, Shimada M, Kurita N, Iwata T, Sato H, et al.. (2012) Survivin expression can predict the effect of chemoradiotherapy for advanced lower rectal cancer. Int J Clin Oncol. 10.1007/s10147-012-0470-0 22936565

[35] Krieg A, Mahotka C, Krieg T, Grabsch H, Muller W, et al (2002) Expression of different survivin variants in gastric carcinomas: first clues to a role of survivin-2B in tumour progression. Br J Cancer 86: 737–743. 11875736 10.1038/sj.bjc.6600153 PMC2375298

[36] Mahotka C, Krieg T, Krieg A, Wenzel M, Suschek CV, et al (2002) Distinct in vivo expression patterns of survivin splice variants in renal cell carcinomas. Int J Cancer 100: 30–36. 12115583 10.1002/ijc.10450

[37] Chang GJ, Rodriguez-Bigas MA, Skibber JM, Moyer VA (2007) Lymph node evaluation and survival after curative resection of colon cancer: systematic review. J Natl Cancer Inst 99: 433–441. 17374833 10.1093/jnci/djk092

[38] Sobin LH, Greene FL (2001) TNM classification: clarification of number of regional lymph nodes for pNo. Cancer. 92: 452. 10.1002/1097-0142(20010715)92:2<452::aid-cncr1342>3.0.co;2-b 11466702

[39] Egger M, Zellweger-Zahner T, Schneider M, Junker C, Lengeler C, et al (1997) Language bias in randomised controlled trials published in English and German. Lancet 350: 326–329. 9251637 10.1016/S0140-6736(97)02419-7

[40] Li F, Yang J, Ramnath N, Javle MM, Tan D (2005) Nuclear or cytoplasmic expression of survivin: what is the significance? Int J Cancer 114: 509–512. 15578717 10.1002/ijc.20768 PMC2829944

[41] Mahotka C, Liebmann J, Wenzel M, Suschek CV, Schmitt M, et al (2002) Differential subcellular localization of functionally divergent survivin splice variants. Cell Death Differ 9: 1334–1342. 12478470 10.1038/sj.cdd.4401091

[42] Hori M, Miki T, Okamoto M, Yazama F, Konishi H, et al (2013) The detergent-soluble cytoplasmic pool of survivin suppresses anoikis and its expression is associated with metastatic disease of human colon cancer. PLoS One 8: e55710. 23405201 10.1371/journal.pone.0055710 PMC3565976

[43] Qi G, Tuncel H, Aoki E, Tanaka S, Oka S, et al (2009) Intracellular localization of survivin determines biological behavior in colorectal cancer. Oncol Rep 22: 557–562. 19639203 10.3892/or_00000471

[44] Li C, Li Z, Zhu M, Zhao T, Chen L, et al (2012) Clinicopathological and prognostic significance of survivin over-expression in patients with esophageal squamous cell carcinoma: a meta-analysis. PLoS One 7: e44764. 23028610 10.1371/journal.pone.0044764 PMC3459962

[45] Zhang LQ, Wang J, Jiang F, Xu L, Liu FY, et al (2012) Prognostic value of survivin in patients with non-small cell lung carcinoma: a systematic review with meta-analysis. PLoS One 7: e34100. 22457815 10.1371/journal.pone.0034100 PMC3311582

[46] Huang LN, Wang DS, Chen YQ, Zhao CL, Gong BL, et al (2012) Expression of survivin and patients survival in non-small cell lung cancer: a meta-analysis of the published studies. Mol Biol Rep 40: 917–924. 23065255 10.1007/s11033-012-2132-8

[47] Zhang T, Fields JZ, Opdenaker L, Otevrel T, Masuda E, et al (2010) Survivin-induced Aurora-B kinase activation: A mechanism by which APC mutations contribute to increased mitoses during colon cancer development. Am J Pathol 177: 2816–2826. 21057000 10.2353/ajpath.2010.100047 PMC2993266

[48] Zhang T, Otevrel T, Gao Z, Gao Z, Ehrlich SM, et al (2001) Evidence that APC regulates survivin expression: a possible mechanism contributing to the stem cell origin of colon cancer. Cancer Res 61: 8664–8667. 11751382

[49] Kim PJ, Plescia J, Clevers H, Fearon ER, Altieri DC (2003) Survivin and molecular pathogenesis of colorectal cancer. Lancet 362: 205–209. 12885482 10.1016/S0140-6736(03)13910-4

[50] Tolcher AW, Quinn DI, Ferrari A, Ahmann F, Giaccone G, et al (2011) A phase II study of YM155, a novel small-molecule suppressor of survivin, in castration-resistant taxane-pretreated prostate cancer. Ann Oncol 23: 968–973. 21859898 10.1093/annonc/mdr353

[51] Cheson BD, Bartlett NL, Vose JM, Lopez-Hernandez A, Seiz AL, et al (2011) A phase II study of the survivin suppressant YM155 in patients with refractory diffuse large B-cell lymphoma. Cancer 118: 3128–3134. 22006123 10.1002/cncr.26510

[52] Cheng Q, Ling X, Haller A, Nakahara T, Yamanaka K, et al (2011) Suppression of survivin promoter activity by YM155 involves disruption of Sp1-DNA interaction in the survivin core promoter. Int J Biochem Mol Biol 3: 179–197. PMC3388737 22773958

[53] Idenoue S, Hirohashi Y, Torigoe T, Sato Y, Tamura Y, et al (2005) A potent immunogenic general cancer vaccine that targets survivin, an inhibitor of apoptosis proteins. Clin Cancer Res 11: 1474–1482. 15746049 10.1158/1078-0432.CCR-03-0817

